# Supplementary material for: One reaction to make highly stretchable or extremely soft silicone elastomers from easily available materials
Source: Nat Commun. 2022 Jan 18;13:370. doi: 10.1038/s41467-022-28015-2 (PMC8766581; doi:10.1038/s41467-022-28015-2)
Supplement: Supplementary file 1 — Supplementary Information [file 41467_2022_28015_MOESM1_ESM.pdf]

Supporting information

One reaction to make highly stretchable or extremely soft silicone elastomers from easily available materials

Pengpeng Hu, Jeppe Madsen, and Anne Ladegaard Skov*

Department of Chemical and Biochemical Engineering, Technical University of Denmark

Søltofts Plads 227, Kgs., Lyngby, 2800, Denmark

E-mail: al@kt.dtu.dk

Theoretical average molecular weight of extended chains

For a platinum-catalyzed reaction system of telechelic Si-H functional PDMS with telechelic vinyl functional PDMS using a small excess of Si-H groups, both the hydrosilylation reaction between Si-H and vinyl groups and the crosslinking of Si-H take place. Assuming the two reactions happen strictly in sequence, the hydrosilylation reaction results in extended chains, which are cross-linked into elastomers by subsequent crosslinking of excess Si-H. According to mass balance equation:

$$(R-1)M_{\mathrm{extended}} = RM_{\mathrm{DMS-H}} + M_{\mathrm{DMS-V}} \qquad \text{Supplementary Equation (1)}$$

where $M_{\mathrm{DMS-H}}$ is the molecular weight of telechelic Si-H functional PDMS, $M_{\mathrm{DMS-v}}$ is the molecular weight of telechelic vinyl functional PDMS, M_{extended} is the average molar mass of the extended network strands and R is the molar ratio of the Si-H to vinyl functional groups. M_{extended} is expressed as:

$$M_{\mathrm{extended}} = \frac{RM_{\mathrm{DMS-H}} + M_{\mathrm{DMS-V}}}{R-1} \qquad \text{Supplementary Equation (2)}$$

Theoretical molecular weight between Si-H groups on intermediate bottlebrush polymers

Assuming crosslinking of Si-H takes place strictly after the full grafting of side chains, molecular weight between Si-H groups on intermediate bottlebrush polymers ($M_{\mathrm{c\_SiH}}$) is expressed as Supplementary Equation (5).

$$f_{\mathrm{brush}} = \frac{R-1}{R}f \qquad \text{Supplementary Equation (3)}$$

$$M_{\mathrm{brush}} = M_{\mathrm{HMS}} + (f - f_{\mathrm{brush}})M_{\mathrm{MCR-V}} \qquad \text{Supplementary Equation (4)}$$

$$M_{\mathrm{c\_SiH}} = \frac{M_{\mathrm{brush}}}{f_{\mathrm{brush}} + 1} \qquad \text{Supplementary Equation (5)}$$

where M_{brush} and f_{brush} are the molecular weight and number of Si-H groups of the intermediate bottlebrush polymer. f and M_{HMS} is the functionality and molecular weight of multi-Si-H functional PDMS. $M_{\mathrm{HMS-V}}$ is the molecular weight of mono-vinyl functional PDMS.

Calculation of molecular weights of bottle-brush network strands (M_c)

M_c are determined based on the measured shear moduli (G) by:[1]

$$M_c = \frac{\rho R T \emptyset^2}{G} \qquad \text{Supplementary Equation (6)}$$

where ρ is the density of silicone elastomer. R is the gas constant. T is the absolute temperature. $\emptyset$ is the gel fraction of elastomers. G is the shear modulus of elastomers.

Supplementary Tables and Figures

Supplementary Table 1. Integration of ^{1}H NMR spectra of a telechelic Si-H functional PDMS (DMS-H11) and its reaction products after heating at 100℃ for 48 h under dry N_2, wet N_2 and dry air conditions, respectively.

**Sample**	**5.10 ppm to 4.40 ppm**	**3.60 ppm to 3.40 ppm**	**2.40 ppm to 2.10 ppm**	**0.50 ppm to -0.40 ppm**
	Si-*H*	SiOCH_2Si	Si-O*H*	CH_3 except at the two ends
DMS-H11	196	0	0	10000
Dry N_2	190	0	0	10000
Wet N_2	132	0	6.37	10000
Dry air	92.2	1.04	4.61	10000

Supplementary Table 2 Integration of ^{29}Si solid state NMR spectra of a telechelic Si-H functional PDMS (DMS-H11) and solid elastomers prepared under normal air conditions.

**Sample**	**9.08 ppm to 6.20 ppm**	**-6.67 ppm to -8.08 ppm**	**-21.64ppm to -23.44 ppm**	**-62.92 ppm to -65.33 ppm**
	$(CH_3)_2CH_2$*Si*O	*Si*-H	*Si*O*Si*	CH_3SiO_3
DMS-H11	0	19.56	100	0
Ela_DMS-H11	1.44	0	100	0.97
Ela_DMS-H21	0.43	0	100	1.19

Supplementary Table 3. Gel fractions and swelling ratios of the investigated networks

Samples	Gel fractions (%)	Swelling ratio
Ela_DMS-H21_DMS-V22_R1.05	67±2	98±13
Ela_DMS-H21_DMS-V22_R1.10	73±1	47±4
Ela_DMS-H21_DMS-V22_R1.15	83±1	26±4
Ela_DMS-H25_DMS-V25_R1.05	69±1	128±17
Ela_HMS-064_MCR-V21_R1.05	73±1	30±2
Ela_HMS-064_MCR-V21_R1.20	78±1	20±1
Ela_HMS-064_MCR-V21_R1.50	83±1	15±3
Ela_HMS-064_MCR-V25_R1.05	52±1	48±2
Ela_DMS-H21	93±1	9±1
Ela_DMS-H25	93±1	12±2
Ela_DMS-H31	88±1	14±2
Ela_HMS-064	88±1	13±2
Ref_DMS-V25	97±0	12±1

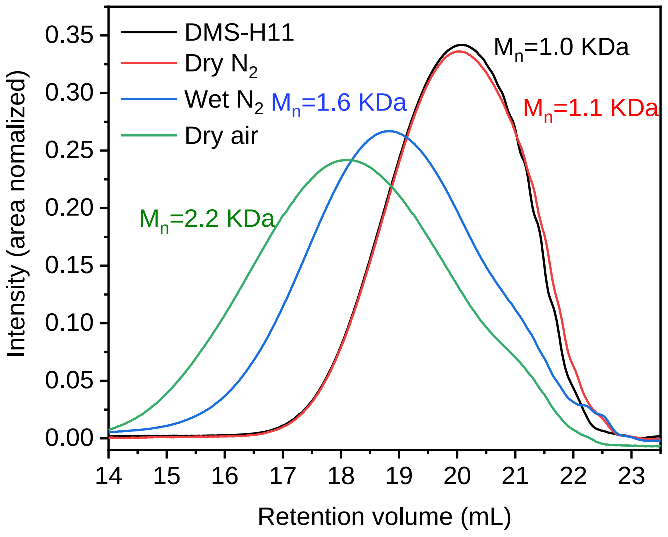

Supplementary Fig. 1. SEC curves of starting polymer DMS-H11 and its liquid products after heating at 100°C for 48 h under dry N_2, wet N_2 and dry air atmospheres, respectively.

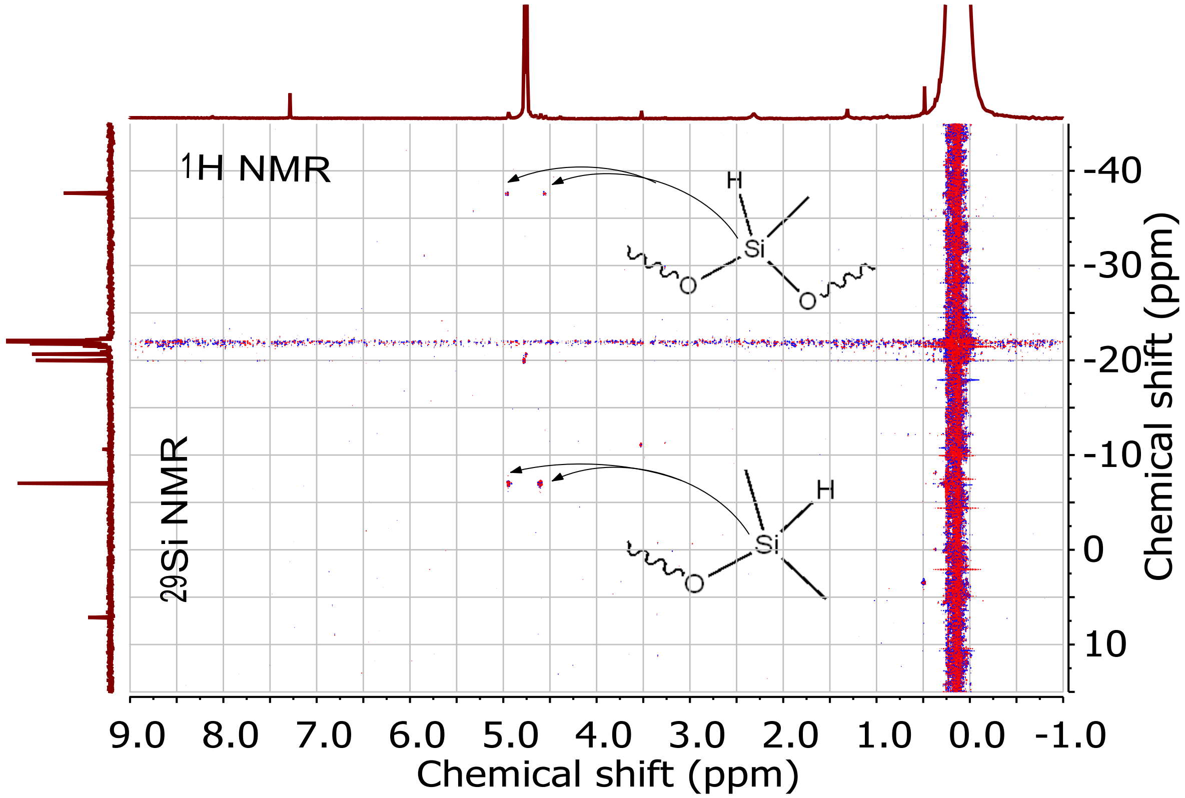

Supplementary Fig. 2. 2D NMR spectrum (^{1}H-^{29}Si NMR coupling) of DMS-H11´s reaction product after heating at 100°C for 48 h under dry air condition.

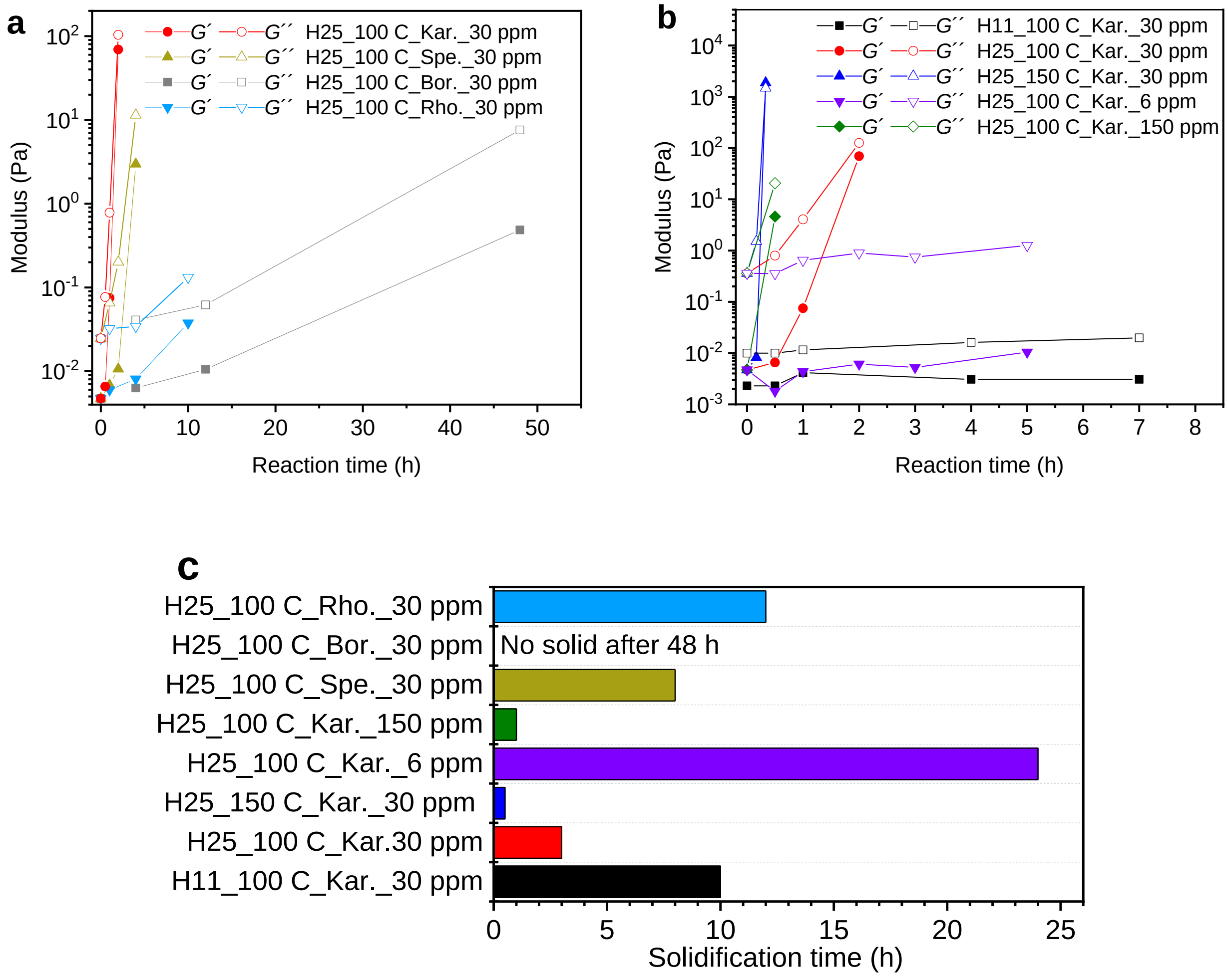

Supplementary Fig. 3 Curing rates for telechelic hydride functional PDMS under various conditions. (a) and (b): Storage moduli (G´) and loss moduli (G´´) of reaction products over reaction time. The samples were sheared with a fixed 2% strain at 0.1 Hz. Specifically, (a) reactions of precursor polymer DMS-H25 are catalyzed at 100 ℃ by various catalysts with 30 ppm concentration, i. e. Karstedt´s catalyst (Kar.), Speiers´s catalyst (Spe.), tris(pentafluorophenyl)borane catalyst (Bor.), and tris(dibutylsulfide) rhodium trichloride catalyst (Rho.). (b) The reactions are catalyzed by Karstedt´s catalyst under different Si-H concentrations (2.0 mol/kg Si-H in precursor DMS-H11 and 0.14 mol/kg Si-H in precursor DMS-H25), reaction temperatures (100 ℃ and 150 ℃) and catalyst concentrations (6 ppm, 30 ppm and 150 ppm). (c) Solidification times for telechelic hydride functional PDMS under various conditions.

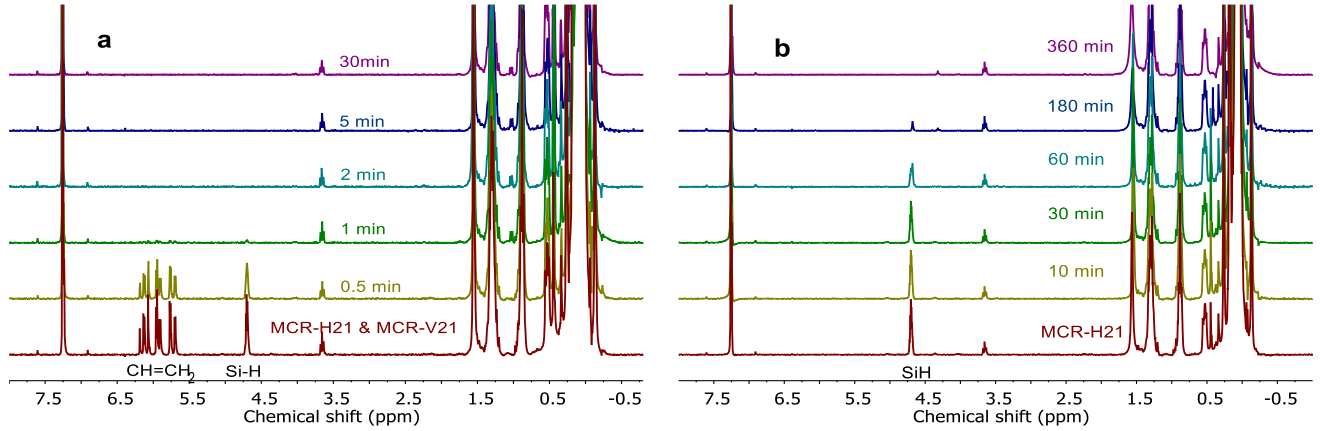

Supplementary Fig. 4. Evolutions of ^{1}H spectra over time for (a) the reaction of mono-Si-H functional PDMS, and (b) hydrosilylation reaction between mono-Si-H functional PDMS with mono-vinyl functional PDMS.

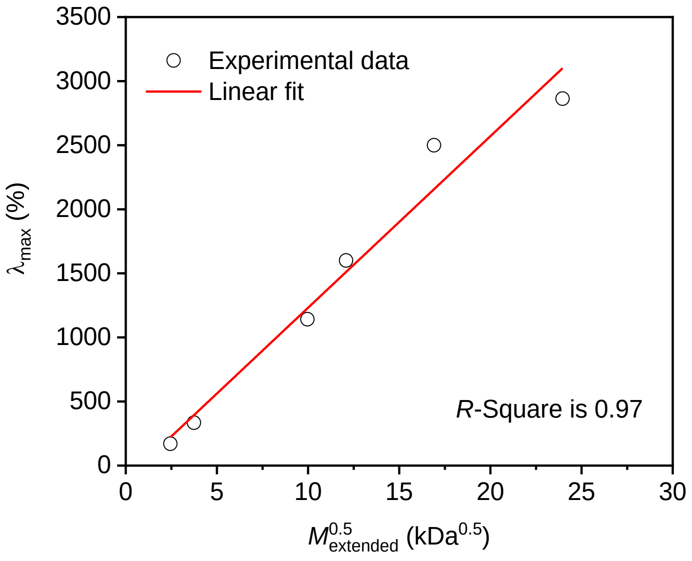

Supplementary Fig. 5. Linear fitting of $\lambda_{\max}$ and $M_{\text{extended}}^{0.5}$. For highly stretchable silicone elastomers, M_{extended} is the theoretical molar mass of precursor polymer calculated by Equation S2. For conventional silicone elastomers Ref_DMS-V25 and Ref_DMS-V41, M_{extended} approximately equals the molecular weight of precursor polymers DMS-V25 and DMS-V41, respectively.

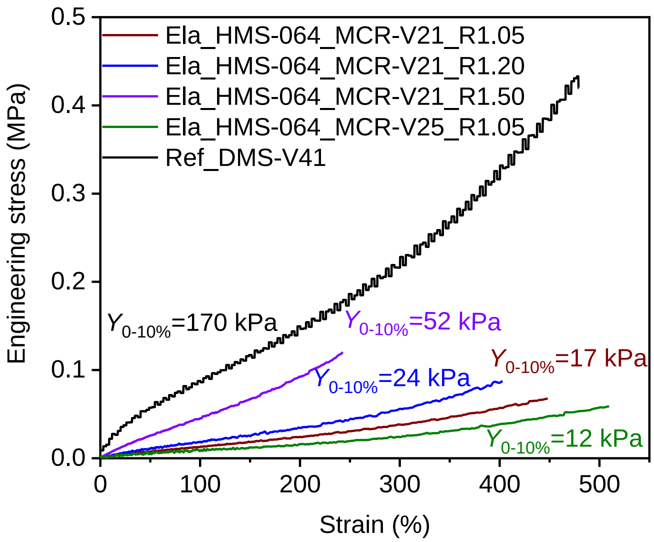

Supplementary Fig. 6. Uniaxial stress-strain curves of extremely soft silicone elastomers and a conventional silicone elastomer.

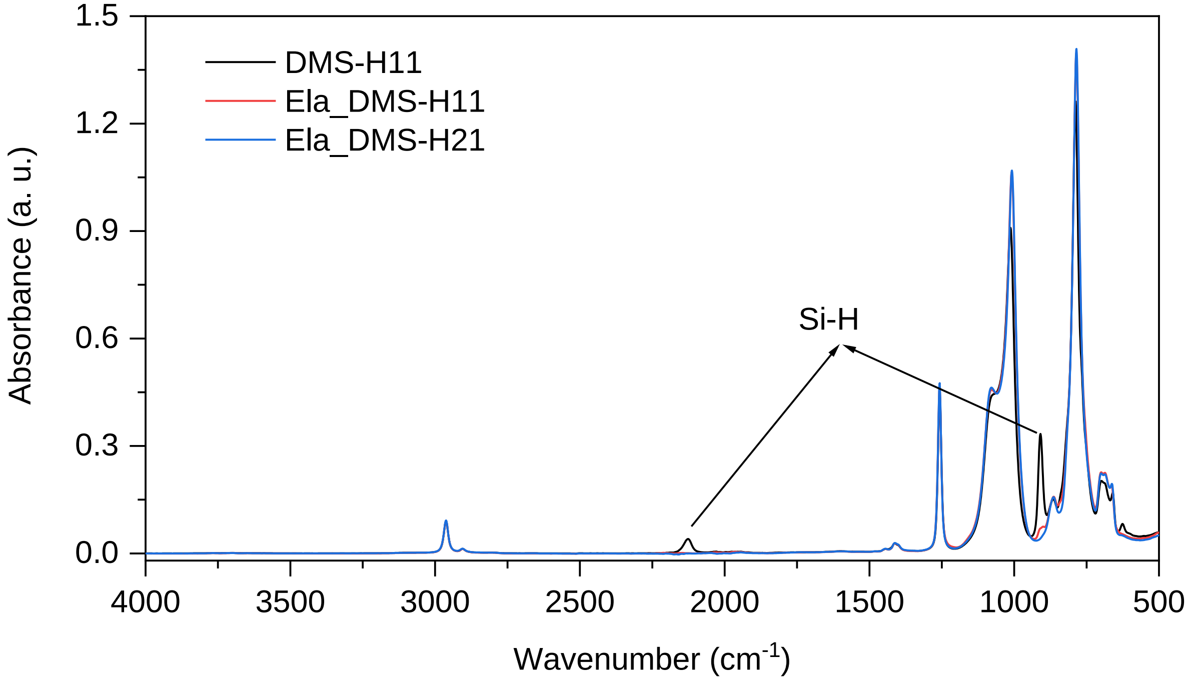

Supplementary Fig. 7. Fourier-transform infrared spectroscopy (FTIR) of elastomers (Ela-DMS-H11 and Ela-DMS-H21) and a precursor polymer (DMS-H11)

Supplementary Reference

(1) Vasiliev, V. G.; Rogovina, L. Z.; Slonimsky, G. L. Dependence of Properties of Swollen and Dry Polymer Networks on the Conditions of Their Formation in Solution. *Polymer* **26**, 1667–1676 (1985)
